# Could magnetic resonance provide *in vivo* histology?

**DOI:** 10.3389/fgene.2013.00298

**Published:** 2014-01-13

**Authors:** Marco Dominietto, Markus Rudin

**Affiliations:** Institute for Biomedical Engineering, University of Zurich and ETH ZurichZurich Switzerland

**Keywords:** *in vivo*, histology, MRI, tumor, classification, physiology, metabolism, tissue

## Abstract

The diagnosis of a suspected tumor lesion faces two basic problems: detection and identification of the specific type of tumor. Radiological techniques are commonly used for the detection and localization of solid tumors. Prerequisite is a high intrinsic or enhanced contrast between normal and neoplastic tissue. Identification of the tumor type is still based on histological analysis. The result depends critically on the sampling sites, which given the inherent heterogeneity of tumors, constitutes a major limitation. Non-invasive *in vivo* imaging might overcome this limitation providing comprehensive three-dimensional morphological, physiological, and metabolic information as well as the possibility for longitudinal studies. In this context, magnetic resonance based techniques are quite attractive since offer at the same time high spatial resolution, unique soft tissue contrast, good temporal resolution to study dynamic processes and high chemical specificity. The goal of this paper is to review the role of magnetic resonance techniques in characterizing tumor tissue *in vivo* both at morphological and physiological levels. The first part of this review covers methods, which provide information on specific aspects of tumor phenotypes, considered as indicators of malignancy. These comprise measurements of the inflammatory status, neo-vascular physiology, acidosis, tumor oxygenation, and metabolism together with tissue morphology. Even if the spatial resolution is not sufficient to characterize the tumor phenotype at a cellular level, this multiparametric information might potentially be used for classification of tumors. The second part discusses mathematical tools, which allow characterizing tissue based on the acquired three-dimensional data set. In particular, methods addressing tumor heterogeneity will be highlighted. Finally, we address the potential and limitation of using MRI as a tool to provide *in vivo* tissue characterization.

## INTRODUCTION

Imaging in diagnosis of suspected neoplastic lesion faces two basic problems: detection and identification of a tumor mass. Detection is based on achieving sufficient contrast (i.e., contrast-to-noise ratio) to enable discrimination of pathological from adjacent normal tissue. Critical factors are high SNR (signal-to-noise ratio) and high soft-tissue contrast, i.e., different tissues should be reflected by different intensity levels in the images and with high spatial resolution. Identification is more demanding and today still based in histological analysis, which faces however, some limitations. Histology is typically carried out on biopsy samples, which provide only focal information on a heterogeneous mass. Sample collection constitutes a burden for the patient and may not always be feasible. Furthermore, longitudinal analyses are difficult. On the other hand, histology yields unambiguous information critical for diagnosis that is based on cellular morphology or on the expression of a characteristic molecular signature expressed by the tissue. The possibility to simultaneously analyze multiple tissue parameters is essential for the identification of the tumor type.

Non-invasive imaging for tumor diagnosis offers unique advantages: minimal burden of the patient, full three-dimensional sampling of the heterogeneous lesion, dynamic measurement of physiological and metabolic processes complementing morphological information, and the possibility for longitudinal examinations. Yet, current imaging approaches are based on structural and physiological phenotypic readouts, which are sufficient for lesion detection and monitoring disease progression or therapy response, but most likely, will not allow identifying the lesion type. Analogous to histological tissue characterization it would be important to assess (a) molecular and cellular characteristics and (b) multiple complementary tissue features in order to achieve a high discriminative power.

As we will see later, the use of complementary imaging modalities that probe different aspects of the pathology would be most promising. Nevertheless, we will focus our current discussion on magnetic resonance based techniques, which are attractive as they provide high spatial resolution, unique soft tissue contrast, a temporal resolution sufficient for studying dynamic processes, and moreover are characterized by high chemical specificity, a feature that is extensively used for chemical and biochemical structure elucidation. In addition, the method can be easily translated into the clinics.

### TISSUE CHARACTERIZATION BY MAGNETIC RESONANCE

Magnetic resonance images represent a weighted distribution of protons (^1^H) in tissue, the predominant source of the signal being tissue water and lipids (adipose tissue). Obviously the signal is proportional to the density of protons in the respective tissue. The weighting function is governed by the proton magnetic properties, which are affected by their local environments due to magnetic and chemical interactions which depend on the nature of tissue (Weishaupt et al., 2006). The effect of the environment on the MRI signal is lumped into parameters describing three distinct relaxation processes (Mark Haacke, 1999): (1) the longitudinal relaxation characterized by the relaxation time T1, which describes the interaction of the spin with its environment, hence the expression spin-lattice relaxation as a crystal lattice constituted the environment in early solid state physics nuclear magnetic resonance (NMR) experiments. T1 relaxation is based on energy exchange between the spin under investigation and its environment and occurs such that the system is driven back to its thermal equilibrium state. (2) Transverse relaxation, characterized by the relaxation time T2 that describes the interaction of the spin under interrogation with neighboring spins, hence the term spin-spin relaxation. T2 relaxation is based on dipole–dipole interactions between spin pairs that fluctuate with regard to their spatial alignment and hence is of stochastic nature. It leads to the irreversible loss of phase coherence and hence to a loss in signal intensity. (3) T2* relaxation is related to T2 and in addition to spin–spin interactions is governed by inhomogeneities in the local magnetic field, e.g., due to difference in magnetic susceptibility between tissues. This local field inhomogeneities are static and hence deterministic and can be accounted for when tailoring the MRI data acquisition (so-called spin-echo experiments). Nevertheless, T2* provides an additional source for contrast. Additional parameters that influence the modulate the interaction of the MRI signal with the environment and hence the MRI signal intensity are molecular diffusion, as well as mechanism leading to coherence/polarization transfer such as chemical exchange reactions or spin diffusion.

Relaxation processes can be influenced by administration of contrast agent, which are either paramagnetic (gadolinium based) or superparamagnetic agents (iron-oxide based). These agents contained unpaired electrons with a strong effect on the local magnetic field that is experience be nearby protons. The contrast mechanism of the two classes of agents is different, yet a detailed description is beyond the scope of this article (Rudin, 2005a). In the context of our discussion it suffices to state that paramagnetic agents enhance the longitudinal relaxation rate, i.e., they reduce T1, while superparamagnetic agents predominantly enhance the transverse relaxation rate, i.e., reduce T2. Apart from enhancing the contrast in static MR images to improve discrimination of distinct tissues, MRI allows monitoring dynamic changes following the contrast agent administration. The contrast change measured in a volume element (voxel) is proportional to the amount of contrast agent in this voxel, which by itself depends on the biodistribution (including compartments within a tissue) and pharmacokinetic properties of the agent. Such dynamic studies yield information on tissue perfusion, vascular leakage, or distribution volumes.

The magnetic resonance phenomena are not only restricted to the detection of protons of water and lipid molecules in tissue. Essentially all magnetic nuclei give rise to signal. The resonance frequency of a nucleus depends on its identity (characterized by the so-called gyromagnetic ratio) and its chemical environment. It is in particular the fact that the magnetic resonance sensitively probes the chemical structure to which the interrogated nucleus is attached that has made the method indispensable for chemical structure elucidation. The identification of a molecular entity is based on the detailed spectral analysis of its resonance frequencies. Translating these approaches to *in vivo* tissue characterization therefore bears considerable potential to enable a detailed (molecular) tissue characterization, which might be of high diagnostic value. Apart from protons, other nuclei such as phosphorus-31, carbon-13, constituents of many biologically relevant molecules are of interest for *in vivo* magnetic resonance spectroscopy (MRS). Yet this method suffers from the low intrinsic sensitivity of magnetic resonance, as these metabolites are typically present at millimolar to sub-millimolar concentration compared to water protons with tissue levels of approximately 80 M.

## PHENOTYPIC TUMOR CHARACTERIZATION

If compared to healthy organs, tumor tissues present in general highly heterogeneous and chaotic architecture. Such heterogeneity is primarily due to the genetic instability of tumor cells that is responsible of the apparently chaotic tumor development, which is reflected in tissue architecture, tumor vasculature, host infiltrates, and metastasis formation (Heppner, 1984; Marusyk et al., 2012). This chaotic behavior occurs at a molecular, cellular, and microdomain level and determines also the interaction with the host environment. The result is the formation of different regions inside the tumor, which may exhibit completely different physiological behavior (Denysenko et al., 2010; Huse et al., 2013).

In order to rationalize the complexities of neoplastic disease, Hanahan and Weinberg (2000) have defined six phenotypic hallmarks of cancer, which correspond to six biological features acquired during tumor development. Those include sustained proliferative signaling, evasion of effects of growth suppressor, resistance to cell death program, acquisition of replicative immortality, development of a vascular network (angiogenesis), invasion of adjacent healthy tissue, and the formation of distant metastases. In a recent publication (Hanahan and Weinberg, 2011), these initial six hallmarks were complemented by four additional features related to the specific behavior of tumor tissue: genome instability, inflammation, reprogramming of energy metabolism, and evasion of immune surveillance.

An important aspect of tumor is that they are not only composed of cancer cells but contain a variety of host derived cells such as immune cells, endothelial cells, pericytes, fibroblasts, stem, and progenitor cells that characterize the hallmarks traits and constitute the tumor microenvironment (Swartz et al., 2012).

Considerable efforts have been invested to assess these tumor hallmarks non-invasively using imaging. Today, methods are available to study tumor proliferation (DNA, protein, and membrane synthesis) using PET and MRI methods, aspects of tumor metabolism using PET and MRS, aspects of tumor vessel architecture and physiology (MRI), apoptotic processes using PET, MRI, and fluorescence imaging, as well as of the invasive potential and propensity for metastasis formation using PET and fluorescence imaging. Yet, all these phenotypic readouts are not specific enough for an unambiguous identification of the tumor type, which is based on unique molecular markers. Secondly, many of these tools are still in an early experimental stage and will not be available in a clinical setting soon.

### TUMOR MORPHOLOGY

Damadian (1971) reported on the observation that T1 relaxation times in tumors are higher than in the adjacent normal tissue and suggested that this feature might be used for tumor detection. This constituted one of the prime motivations that later led to the development of MRI. Nowadays, modern MRI scanners offer several tools for detecting and characterize tumor.

#### Detection of tumors based on altered relaxivity values

Despite the fact that the basic biophysical mechanism leading to tissue specific relaxivity values are poorly understood, the evaluation of relaxivity parameters are of high diagnostic value.

**FIGURE 1:**
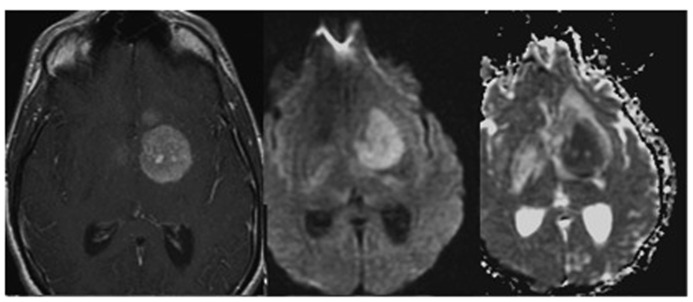
**T1-weighted image of a glioma following contrast enhancement using a gadolinium-based contrast agent (left). Diffusion weighted images DWI (middle**), and apparent diffusion coefficient map ADC (**right**) of the same tumor patient. Adapted from Young (2007), reproduced with permission.

According to the type of MR sequence and the relative parameters, it is possible to acquire a signal, which is mostly dominated by one of these contributions. Most established are T1-weighted, T2-weighted or proton density weighted images (Haacke et al., 1999). By optimizing the contrast between neoplastic and normal tissue it is in generally possible to detect the cancer lesion, to identify sub-regions displaying different tissue characteristics (dense versus non-dense tissue, poorly versus highly vascularized, necrotic areas, edematous tissue, etc.), and to monitor of tumor progression or regression. Yet, these phenotypic measurements are in general not sufficient for “histological” classification of the tumor. Instead some generic tissue features are reflected. For example, T1-weighted images are usually used to assess the gross morphology of the tumor as shown in **Figure 1** (left). As rule of thumb, regions with high water content appear dark, while regions with high fat content appear bright (Weishaupt et al., 2006). In combination with gadolinium-based contrast agent such as Gd-DTPA it is possible to assess regions displaying high uptake of the agent indicative of hemorrhage and leaky vessels. Areas, for which little uptake is observed are commonly associated with necrotic or edematous domains. Only when waiting sufficiently long these areas will accumulate extravasated contrast agent via passive diffusion.

In T2-weighted images areas with high water content appears bright. Since most diseases are characterized by increased water content in tissues associated with an inflammatory tissue response, T2-weighted are particularly useful for pathological investigation. Dark regions may indicate high blood content such as hemorrhage, vessels, or angiomas.

In proton weighted images (Westbrook, 2010), bright areas indicates high proton density tissue, such as cerebrospinal fluid or edema, while dark areas indicate low proton density such connective tissue (i.e., tendons) or cortical bone.

Nowadays, tumor detection based on altered T1 and T2 relaxivity values is commonly used to diagnose and follow-up different kinds of tumor comprising, among the others, brain tumor (Young, 2007), breast tumor (Heywang-Kobrunner et al., 1997), prostate cancer (Verma et al., 2012), and gastric cancer (Wang et al., 2000). By means of T1 and T2 weighted images and in combination with contrast agent, as Gd-DTPA or superparamagnetic nanoparticles, it is possible to assess tumor morphology and grossly identify edematous and necrotic regions. Moreover, kinetics and extent of contrast agent uptake are considered as an indicator of prognostic quality.

The possibility to obtain high-resolution and high-contrast images of soft tissue with similar density but different relaxivity values makes MRI the method of choice for the detection of solid tumors.

#### Alteration in cellularity: measuring the apparent diffusion coefficient

Diffusion Weighted Imaging (DWI) measures the random movement of the water molecules and allows deriving the so-called apparent diffusion coefficient (ADC) for each voxel (Haacke et al., 1999). “Apparent” since the measured coefficient corresponds to a weighted average across individual diffusion coefficients for all compartments contained in this voxel. Also, structural barriers like cell membranes, or perfusion effects affect diffusion (Haacke et al., 1999). Given this definition and the fact that the diffusion coefficients within cells and in the extracellular space are different, with D_intracellular_ < D_extracellular_, it becomes apparent that the ADC values are sensitive to the relative size of these two compartments. Hence, regions with densely packed cells will show low ADC values. This has been exploited in the characterization of brain neoplasms. High grade tumor neoplasms display significant reduction of ADC and correspondingly a higher signal in DWI as compared to lower grade (Okamoto et al., 2000; **Figure 1** middle and right). Fluid filled cysts or edematous regions appear hyperintense in ADC maps (and hypo-intense in DWI) when compared to the normal parenchyma because they largely correspond to bulk water enabling unrestricted diffusion (within the MRI timescale; Drevelegas and Papanikolaou, 2011).

#### Inflammatory status: edema formation and infiltration of immune cells

Recent data have expanded the concept that inflammation is a critical component of tumor progression (Coussens and Werb, 2002). The quantification of the inflammatory status is crucial in the determination of the tumor volume, since its value is an important prognostic factor with regard to the treatment of malignant tumors (Xie et al., 2005). Moreover, inflammation may also influence therapy outcome in two opposite ways, in particular for brain tumors such as gliomas (Kleijn et al., 2011). It can lead to tumor control, by killing cancer cells and establishing anti-cancer immunity, or it may further promote tumor growth, by participating in glioma reoccurrence and progression. It is therefore evident that the possibility to monitor the inflammation status *in vivo*, i.e., by monitoring immune cells, is a crucial step in tumor management. Traditionally, such evaluation is performed *ex vivo* using cytometry and immunohistochemistry methods, or *in vivo* using labeled-radionuclides for PET (Positron Emission Tomography) or SPET (Single Photon emission tomography) scanner (Ahrens and Bulte, 2013). However, recent developments, in particular the possibility to prepare non-toxic MRI probes for cell labeling, enables MRI based tracking of immune cells. Compared to PET or SPET, MRI has the advantages that it does not use ionizing radiation and provides higher spatial resolution.

**FIGURE 2:**
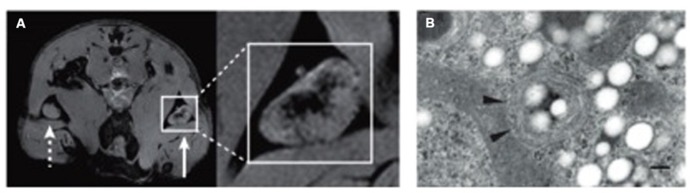
**Example of tracking immune cells with MRI using SPIO nanoparticles and PFC emulsions. (A)** Imaging of *in vivo* antigen capture and trafficking of dendritic cells (DCs). Sentinel DCs were labeled *in situ* by intradermal injection of unlabeled (**dashed arrow**) or SPIO-labeled (**solid arrow**) irradiated cancer cells, which function as a vaccine. Following phagocytosis of both SPIO particles and tumour antigens in a process known as magnetovaccination, the hypointense DCs migrate into the medulla of the draining popliteal lymph node. **(B)** An electron micrograph of a perfluorocarbon (PFC)-labeled DC is shown. Numerous bright spots (PFC droplets) are observed inside the cell. Particles appear as smooth spheroids (Ogawa et al., 1990). Arrowheads indicate vesicles. The scale bar represents 200 nm. Adapted from Ahrens and Bulte (2013), reproduced with permission.

Magnetic resonance imaging (MRI) cell tracking involves exogenous cell labels such as iron oxide nanoparticles, perfluorocarbon (PFC) nanoemulsion, or genetically encoded MRI reporters (Ahrens and Bulte, 2013; **Figure 2**). Immune cells can be labeled with superparamagnetic iron oxide based (SPIO) nanoparticles in two ways: (i) by *ex vivo* labeling of harvested cells that are incubated with SPIO nanoparticles in media typically using a transfection agent, or (ii) by non-selective *in situ* labeling of the phagocytic cells, such as macrophages, following intravenous injection of SPIO nanoparticles (Bhakoo et al., 2006). PFC emulsion can be used to track cells using the same labeling strategies. PFC-based cell tracking provides high specificity for cell detection (i.e., a high signal-to-background ratio can be achieved as there is no endogenous source of a fluorine signal) and enables the quantitative measurements of the amount of cells. Yet they require a specific MRI coil tuned to the resonance frequency of ^19^F nuclei. Disadvantages of using passive labeling strategies are that only the presence of the label is detected, which is not necessarily identical with the presence of cells. Cells may release the label into the environment, e.g., after death, yielding to a false positive signal. Also, the presence of the label does not yield any information on the status of the cell, i.e., whether it is alive or dead. Finally, for dividing cells (which is not relevant for the immune cells) the label will be subsequently diluted. In addition, a passive label will be degraded over time. Genetic encoded reporters avoid some of these issues. They only yield a signal when the gene is expressed, i.e., when the cell is alive, and the presence of labels also indicates the presence of the cell. On the other hand, the sensitivity of genetic cell marking is in general inferior to that of potent exogenous labels.

Magnetic resonance imaging cell tracking can also be used to monitor inflammation related to other disease as neurological disorders, autoimmune diseases, or transplant rejection. Moreover, it is likely to become an important tool also in cell therapy (i.e., stem cells for different diseases) with the specific aim to guide cell injections and subsequently monitoring their migration (Bulte, 2009; Hong et al., 2010).

One of the consequences of the inflammatory status is the formation of a peritumoral edema which is the results of several cellular mechanism (Stummer, 2007). Although its prognostic value for diagnosis, as well its role in the course of disease is still a matter of discussion, peritumoral edema may cause severe neurological symptoms in case of brain tumor, and remains a challenge in the treatment of glioblastoma patients (Kleijn et al., 2011; Stummer, 2007).

The evaluation of edema by means of MRI is usually performed using T2-weighted sequences that are quite sensitive to water content, and by assessing changes in ADC. The regions affected by edema are characterized by prolonged T2 values and therefore appear hyperintense in T2-weighted images.

### TUMOR PHYSIOLOGY

The physiology of tumor tissues is directly dependent on the structure and functionality of the vascular network developed during tumor growth. The newly formed vessels are responsible for the delivery of the nutrients from the hosting tissue to the tumor and for the removing of waste metabolites from the tumor. Characterization of the angiogenic process is therefore essential either for understanding the chaotic steps of tumor evolution or for the development of anti-angiogenic drugs (Marmé and Fusenig, 2007).

Tumor vasculature deviates profoundly from that of the normal organs both in vascular architecture and functionality. The vascular network of solid tumor does not show the hierarchical branching patterns characteristic for the majority of healthy organs. This is the results of the opportunistic nature of the angiogenic process, which in tumor seems not to follow physiological pre-determined steps (Tropres et al., 2001; Kiselev et al., 2005). Initially avascular tumor masses trigger the development of new angiogenic vessels as a consequence of hypoxia and the secretion of angiogenic factors (Lemasson et al., 2013). Alternatively, tumors may grow along one or more existing vessels and co-opt them in the tumor structure in a parasitic manner. In both cases vessels usually remain in a primitive status with immature vascular walls and proper support by the tissue matrix. Tumor vascular networks therefore consist of tortuous micro-vessels exerting chaotic branching, arterial-venous shunts, and are subject to acute or transient collapse (Heywang-Kobrunner et al., 1997).

The lack of maturation of the primitive vessel network gives origin to a few abnormalities in vascular function. Tumor capillaries show high permeability compared to the healthy ones (Tropres et al., 2004). This results in a profound extravasation of erythrocytes and plasma in the adjacent tissue leading to an elevated interstitial fluid pressure and to a rise in the viscous resistance to blood flow (Dominietto, 2012). Second, because of this resistance and chaotic structure, the blood circulation or perfusion within such vessels is rarely correlated to the metabolic demands of solid tumor (Heywang-Kobrunner et al., 1997). Moreover, the clearance of metabolites from the tissue and the drainage by the venous system do not work properly and are responsible of the accumulation of blood in the tumor tissue.

To complicate matters even more, the degree of abnormalities changes in different kinds of tumors and also during different stages of the same tumor. While from a biological point of view the origin of these physiological fluctuations is poorly understood, the assessment of vascular abnormalities constitute an attractive biomarker, as it clearly distinguishes neoplastic from normal tissue. Various structural and physiological aspects of tumor vasculature can be quantified by MRI and used for classification and staging of tumors.

### NEOANGIOGENESIS: VASCULAR STATUS AND PHYSIOLOGY

**FIGURE 3:**
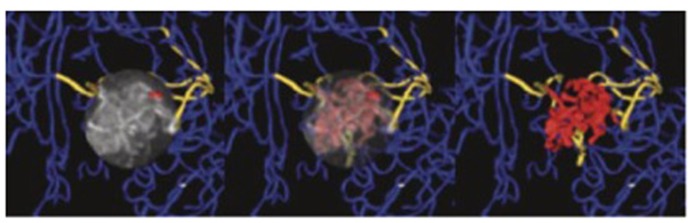
**Magnetic resonance angiography of a brain tumor to evaluate the tortuosity of the vascular network.** Vessels within the tumor nidus are shown in red, vessels supplying or passing through the nidus in gold, while normal vessels outside the nidus are blue. The nidus, containing type II tortuosity vessels, is volume rendered at full opacity (**left**), at partial opacity (**center**), while vascular structures exclusively are shown at (**right**). Adapted from Bullitt and Gerig (2003), reproduced with permission.

The vascular network of bigger vessels (diameter > 50 μm) can be directly visualized by means of magnetic resonance angiography (MRA) technique as shown in **Figure 3**. Three different methods are currently available: (a) time-of-flight (TOF), (b) contrast enhanced (CE), and (c) phase contrast (PC) MRA. All these approaches aim at generating a high contrast between the vascular lumen (blood compartment) and the surrounding tissue to enable the segmentation and extraction of vascular structures.

Time-of-flight angiography (Heverhagen et al., 2008) exploits the intrinsic differential behavior of protons in flowing blood as compared to stationary tissue and does not require the administration of contrast agents. Briefly, by a combination of radiofrequency excitation pulses all the spins of the excited volume will be saturated and, because of that, the signal will be largely suppressed. However, blood that has entered the imaged volume, will give rise to the full signal intensity, as it has not experienced previous saturation. Whether a vessel can be depicted using TOF-MRA depends on whether it can be reached by fresh blood during excitation.

Contrast enhanced (Chandra et al., 2012) takes the advantage of the administration as a bolus of a contrast agent in the blood stream during MRI acquisition. Gadolinium based contrast agent will produce an enhancement of the signal in T1-weighted sequences, while iron-based contrast agent will cause dephasing of the nuclear magnets decreasing the overall signal in T2-weighted acquisitions. Acquisition has to be fast enough that extravasation of the contrast agent remains minimal. Angiograms are then obtained by comparing pre- and post-contrast images.

Phase contrast (Thomas and Wells, 2011) utilizes the change in the phase shifts of the flowing protons in the region of interest to create an image. Spins moving along the direction of a magnetic field gradient receive a phase shift proportional to their velocity. This is usually accomplished by applying gradient pairs, which sequentially dephase and then rephase spins during the sequence. Use of phase-sensitive image reconstruction allows depticting the vascular systems exclusively and more over provides information on blood flow velocities.

Despite the high spatial resolution of MRI if compared to other diagnostic imaging modalities, it is not possible to depict the fine details vascular tree as (a) the typical vessel diameter of tumor vessels is in the range 5–50 μm, and (b) flow velocity in these vessels is typical small. Only with high-field magnets and sophisticate coils that are used in experimental studies in animals, enabling an isotropic spatial resolution of the order of 50 μm, it has been possible to depict larger branches of the tumor vasculature (>50 μm) using CE techniques in subcutaneous or orthotopic tumors in mice. Nevertheless, MRI offers the ability to indirectly investigate small vessels by means of a special CE technique called vessel size imaging (VSI).

Vessel size imaging (Tropres et al., 2001; Kiselev et al., 2005) allows the evaluation of the mean vascular density (MVD; Lemasson et al., 2013) and the average vessel diameter (AVD) in a voxel or in a volume (Tropres et al., 2004). The approach is based on the simultaneous measurement of the changes in T2 and T2* induced by the administration of an intravascular super-paramagnetic contrast agent. While T2 depends on the dipolar interaction between the intravascular contrast agent and the tissue protons, which scales to the surface of the vessel T2* effects ar proportional to the bulk effect of the contrast agent to the local magnetic susceptibility, which scales to the vascular volume. From indirect measurements of vessel surface and volume we can infer on the average radius of the vessels in a given region-of-interest.

The dimension and density of the vessels is an important index when studying angiogenesis. When combined with an independent measurement of the tumor blood volume (TBV), it constitutes an index of the organization of the vascular network. Identification of vessels of various diameter (from big to small) indicates a hierarchical network, while the presence of only small vessels is an index of the poor organization of the vascular tree.

While information on the vascular architecture within the tumor is a downstream manifestation of the angiogenic process, it is important to derive physiological information in order to understand the implication on substrate delivery, which essentially determines the fate of the tumor. Capillary vessels like arterioles and venules are permeable to the substances present in the blood to enhancing compound exchange between the blood and tissue compartment. It has been shown that in tumors also relatively big vessels are highly permeable due to the immature structure of the vascular wall. This results on an almost completely leaky network with a highly non-uniform blood supply to tumor tissue (Dominietto, 2012).

The characteristically high permeability of tumor vessels has been suggested as biomarker for angiogenesis (Feng et al., 2008), and for evaluating antiangiogenic treatment efficacy (Alic et al., 2011; O’Connor et al., 2011; Najafi et al., 2012). Vascular permeability values are commonly assessed by means of T1-weighted dynamic contrast enhanced (DCE) acquisitions, involving serial images of the same region during the administration of a gadolinium-based contrast agent (Rudin et al., 2005). The measured MRI signal enhancement curve is fitted using a two-compartment model originally proposed by Tofts and Kermode (1991). In its simplest version the model comprises a vascular and an extracellular compartment. Fitting to the enhancement curve is carried out by optimizing two parameters, the vascular permeability defined by the transfer constant *k*^trans^, a measure for the rate of contrast agent extravasation, and the volume of the extracellular compartment *V*_e_.

**FIGURE 4:**
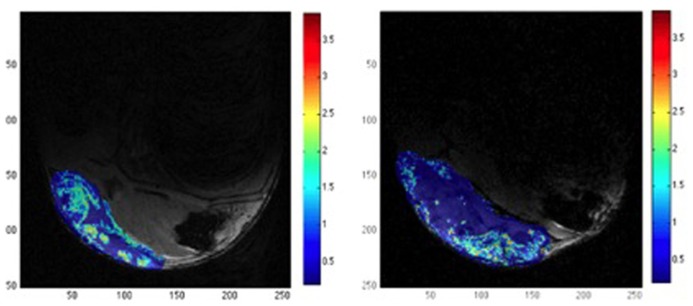
**Example of relative tumor blood volume rTBV (color) overlaid on a structural MR image (gray level).** The images show the effect of DMOG treatment that affects angiogenesis process (**left**) versus placebo (**right**). DMOG treated tumor shows multiple small regions with relative high rTBV, while placebo treated tumor present only one big region with significant rTBV. The color bar indicates the rTBV values in arbitrary units. Adapted from Dominietto et al. (2012), reproduced with permission.

Two other important parameters giving insight into the vessel functionality are tumor blood flow (TBF) and TBV (**Figure 4**). While TBV measure the volume of the vascular compartment in a region-of-interest, TBF assess the exchange of blood within this volume per unit time. Both parameters can be estimated by means of T2*-weighted dynamic susceptibility contrast (DSC) MRI experiments recording the change in signal intensity during the administration of a super-paramagnetic contrast agent (Barbier et al., 2001; Rudin et al., 2005). For data analysis, it is assumed that, due to its nanoparticulate size, the contrast agent remains confined to the blood compartment, at least for the duration of the measurement.

#### Tumor oxygenation

The oxygenation is another important factor in tissue characterization since abnormal oxygen levels have several implications in tumor progression and treatment (Nilesh and Quarles, 2011). In particular, a hypoxic environment is known to promote angiogenesis, inflammatory behavior, genetic instability, invasiveness, and metastasis formation. Hence, hypoxia is associated with increased malignancy and causes reduced efficacy of radio- and chemo-therapy.

**FIGURE 5:**
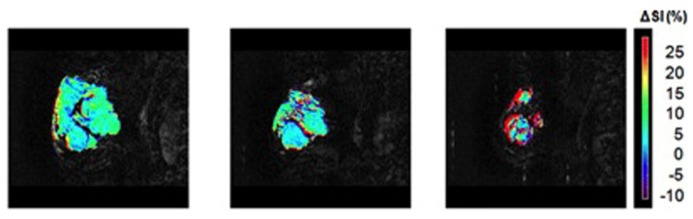
**BOLD MRI for a patient with breast tumor exhibiting a partial response to therapy.** Images show a signal enhancement maps (**color**) overlaid on T2-weighted anatomical images. Images have been acquired 1 week before start of neoadjuvant chemotherapy (**left**), after one cycle of chemotherapy showing small signal response (**middle**) and after four cycles of chemotherapy demonstrating a striking change in tumor characteristics in response to therapy (**right**). Adapted from Jiang and Weatherall (2013), reproduced with permission.

Two MR based techniques have mainly developed to image tissue oxygenation status: BOLD-MRI and fluorine-19 NMR (^19^F-NMR). BOLD (Blood Oxygen Level Dependent; **Figure 5**) contrast assesses alterations in the relative concentrations of deoxyhemoglobin (dHb) and oxyemoglobin (HbO_2_) concentration in blood (Ogawa et al., 1990). The blood oxygen saturation given by the ratio [HbO_2_]/(HbO_2_] + [dHb]) changes according to local cellular activity and hence oxygen consumption. Since dHb is paramagnetic, it induces local changes in magnetic susceptibility, and hence a decrease of T2*, in the region surrounding the vessel. Correspondingly, increased oxygen saturation will lead to an increased signal intensity when using T2*-weighted pulse sequences (Nilesh and Quarles, 2011). This method has been used to monitor treatment response during phototherapy (Gross et al., 2003), upon administration of vasomodulators (Robinson et al., 1995; Taylor et al., 2001), to predict the response radiotherapy response, which is known to critically depend on the oxygenation status of the tumor (Rodrigues et al., 2004), and to characterize vascular architecture in general (Robinson et al., 2003). While BOLD based methods provide accurate qualitative information of blood oxygenation it is difficult to extract reliable quantitative data.

^19^F-NMR approaches involve the administration of PFCs, which are well known for their high oxygen carrying capacity. It has been demonstrated that the ^19^F relaxation time T1 is linearly dependent on oxygen tension (Joseph et al., 1985; Fishman et al., 1989) and with proper calibration it is possible to quantitatively assess tissue oxygenation at equilibrium, or following a metabolic perturbation. However, given the difficulty of delivering sufficient quantities of PFCs to tumor tissue, as many of these agents require intra-tumoral injection, the method has remained a preclinical tool (Nilesh and Quarles, 2011).

#### Acidosis: link to metabolism

Metabolic reprogramming of tumor cells has been recognized already very early. It has been observed that neoplastic tissue exerts high glycolytic activity even under conditions of normoxia (Warburg effect; Gatenby and Gillies, 2004). In fact, measurement of enhanced glucose utilization with PET using [^18^F]-2-fluoro-2-doxyglucose (FDG) as tracer has emerged as important diagnostic tool for tumor diagnosis, in particular for detection of the metastatic burden. Only recently, molecular mechanism underlying this reprogramming, linking metabolic processes to altered gene expression are being elucidated (DeBerardinis et al., 2008; Ward and Thompson, 2012). Glycolysis leads to the production of lactic acid from pyruvic acid via pyruvate dehydrogenase, which is responsible for acidosis. Nevertheless, the intracellular pH of solid tumor, which is the result of a balance between metabolic proton production, proton buffering capacity and transport processes, is maintained within a range of pH = 7.0–7.2 (Zhang et al., 2010). Hence, despite increased acid production, tumor cells maintain a normal slightly alkaline intracellular pH. The major acid load is transported outside the cells but, since the acid cannot be easily removed by the abnormal vasculature, the microenvironment will become acidic (Zhang et al., 2010).

**FIGURE 6:**
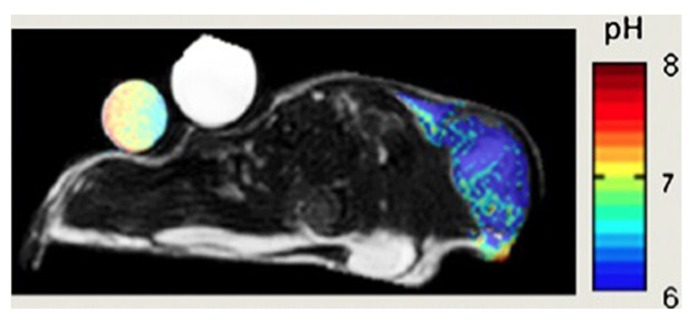
**pH map of mouse MCF-7 breast tumor model.** pH was measured by administration of a paramagnetic CEST (Chemical Exchange Saturation Transfer) MRI using pH-sensitive contrast agent ytterbium-1,4,7,10-tetraazacyclododecane-1,4,7 tetraacetic acid, 10-oaminoanilide. Adapted from Zhang et al. (2010) reproduced with permission.

Tissue acidosis is an important feature of the tumor microenvironment which has been shown to drive local invasion and not surprisingly several approaches have been described to assess tumor pH non-invasively (**Figure 6**). *In vivo* MRI and MRS can be used to measure pH values *in vivo* either using endogenous or exogenous compounds (Raghunand, 2006). MRS methods are generally based on a difference in chemical shifts between pH-dependent and pH-independent resonances (Zhang et al., 2010). A resonance becomes pH dependent when the resonance frequency of the protonated form is distinct from that of the deprotonated form and when the exchange reaction is fast compared to the MRS time scale, which is defined by the frequency difference of the two resonances. Different nuclei can be used to determine tissue pH using this approach: ^31^P (Gadian and Radda, 1981), ^1^H and hyperpolarized ^13^C (Gallagher et al., 2011).

An alternative approach using MRI relies on perturbing the relaxivity of water via pH-dependent relaxation agents. Small molecules Gd-based agents, whose relaxivity is pH dependent, have been recently synthesized (Zhang et al., 1999; Raghunand et al., 2002; Pierre et al., 2006). For the pH quantification, this method requires knowledge of the concentration of the agent in each voxel.

Finally, a new generation of agents that have been developed to generate contrast via chemical shift saturation transfer (CEST) enable pH measurement (Zhang et al., 2010). The dynamic process of CEST can be described by 2-pool chemical exchange model, wherein the magnetization is exchanged between a labile proton (e.g., an amide proton of proteins) and bulk water. The two resonances have to be distinguishable. In the experiment one of the two resonances (the smaller proton pool) is magnetically labeled (saturated) and the transfer of label to the exchange partner (the water proton) is monitored. For example, the resonance of amide protons is saturated and the transfer of saturation to the water resonance, i.e., the decrease of the water signal intensity, is analyzed. Mathematical modeling based on Bloch equations coupled by chemical exchange yields estimates for the exchange rate, which depend on pH. In general, exchange rates are slower at a low pH. There are three main categories of CEST imaging: diamagnetic (Pacheco-Torres et al., 2011), paramagnetic (Liu et al., 2012), and amide proton transfer (Sun et al., 2011).

### TUMOR METABOLISM

**FIGURE 7:**
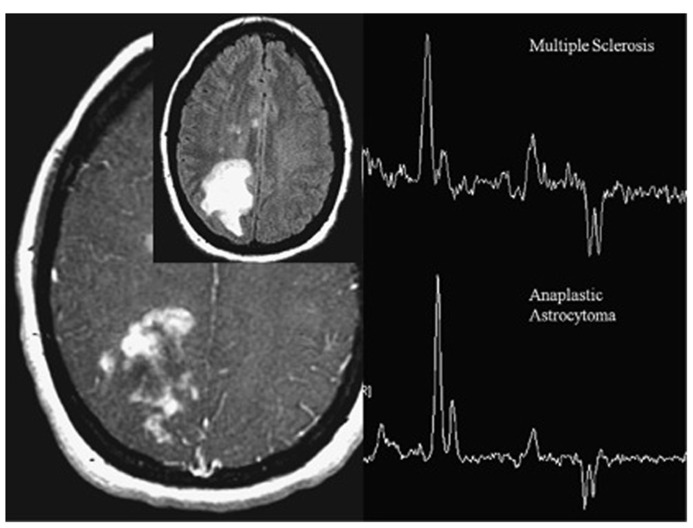
**Magnetic resonance spectroscopy from patient with heterogeneously enhancing white matter lesions.** The indistinguishable spectra demonstrate elevated choline, low NAA, and moderate lactate. One spectrum represents tumefactive multiple sclerosis (MS), the other one anaplastic astrocytoma. In anaplastic astrocytoma, choline elevation reflects membrane synthesis as marker of active proliferation, whereas in MS, it represents membrane injury and degradation of membrane phospholipids. Adapted from Young (2007) reproduced with permission.

The concentration various metabolites can be measured by means of MRS (**Figure 7**). Compounds accessible by MRS relate to the tumor hallmarks deregulated energy metabolism, sustained proliferation, and resisting cell death (Hanahan, 2000). Metabolites related to energy metabolism are the substrate glucose and the intermediates of glycolytic processing including pyruvate and lactate, which can be assessed using either ^1^H or ^13^C MRS. Recently, hyperpolarization techniques such as ^13^C MRS combined with dynamic nuclear polarization (DNP) have been introduce. They enhance the sensitivity of MRI by three to four orders of magnitude, though the lifetime of the hyperpolarized state is typically less than 1 min in biological tissue, which limits the applicability of the method. Nevertheless, it could be shown using DNP ^13^C MRS in addition to glycolytic processing of pyruvate that the label is also transferred to alanine, which indicates the increased anabolic (proliferative) activity of tumors. The prime energy substrate produced by anaerobic and aerobic glucose processing is adenosine-triphosphate (ATP), which can be assessed, together with other phosphorus containing metabolites such as phosphocreatine (PCr), nicotinamide adenine dinucleotide phosphate (NADP), or orthophosphate (HPO_4_^2^^-^/ H_2_PO_4_^-^) using ^31^P MRS. A characteristic of tumors is their acidic environment, which is related to their high glycolytic activity. Intracellular pH is commonly assessed by comparing the resonance frequency of the PCr and HPO_4_^2^^-^/ H_2_PO_4_^-^ resonance. Due to the fast proton exchange (with regard to the MRS time scale) between HPO_4_^2^^-^and H_2_PO_4_^-^ only one resonance signal is observed for the two compounds, the frequency of which depends on the relative concentration of the two and hence sensitive to the pH value. In contrast, the PCR signal does not depend on the pH value. Hence by measuring the frequency difference of the PCr versus the HPO_4_^2^^-^/ H_2_PO_4_^-^ signal, the pH value can be accurately determined (Zhang et al., 2010). High proliferation capacity implies high rates of membrane synthesis. Not surprisingly tumor typically show high levels of phospholipid precursors such as choline/phospho-choline or ethanolamine/phospho-ethanolamine. While the non-phosphorylated compound are typically measured using ^1^H MRS, the phosphorylated analogs are detected as a phosphomonoester resonance using ^31^P MRS. In fact the characteristic nature of this peak has been used to assess therapy response already very early (Ng et al., 1989). In clinical routine, these proliferation readouts are mainly used in the diagnosis and monitoring of brain tumors (Bhakoo et al., 2006; Ahrens and Bulte, 2013). Finally it has been shown that ^1^H MRS of lipid signal may be used to study apoptotic signaling (Schmitz et al., 2005).

The evaluations of all the phenotypic readouts previously described are indirect measurement of processes that occur at a molecular level. Although these readouts provide relevant information on the tumor status, they are of generic nature and may lack the specificity required for the final diagnosis: different molecular processes, for example, can lead to almost identical phenotypes. The identification of tumor types is based on its molecular composition. Hence, similar to the histological analysis imaging, methods have to be developed that provide cellular and molecular information (see Assessing Cellular and Molecular: Molecular Imaging Approaches). Alternatively, we might consider compiling the various structural, physiological and metabolic information collected into a fingerprint that may provide the desired degree of specificity in selected cases (see Mathematical Tools for Handling Multi-Parametric Imaging Data: a Classification Problem).

### ASSESSING CELLULAR AND MOLECULAR: MOLECULAR IMAGING APPROACHES

Final histological tumor diagnosis/classification is based on the expression of specific molecular markers, hence it becomes obvious that whenever non-invasive imaging should reach that stage, it mast yield temporal-spatially resolved information on the expression of such tumor-specific biomolecules, typically surface epitopes. This asks for molecular imaging solutions visualizing molecular targets or molecular processes occurring at the molecular and cellular levels (Martin, 2011). To achieve this goal, exogenous contrast agents coupled with a molecule that targets specific cell receptors or interacts with specific enzyme or proteins *in vivo* are needed. Quantification of results in molecular imaging refers to the ability to estimate the concentration of the exogenous agent that has reached a specific location at a specific time, and in special cases, to estimate the rate of a biochemical process, such as enzymatic cleavage.

Today, there are a considerable number of publications describing target specific compounds tested in *in vitro* assays that have the potential for *in vivo* imaging; yet only few studies are reported with living organism.

Antibody-based imaging agents constitute a large majority of tumor specific probes (Rudin, 2005b). The tyrosine kinase receptor Her-2/neu, for example, is a protein over-expressed on the surface of breast cancer cells, and other human tumors (Slamon et al., 1989). Approximately 30% of mammary carcinomas express this epithelial growth factor receptor. High expression levels correspond to poor prognosis; hence, Her-2/neu may constitute an attractive target for immunotherapeutic agents, such as humanized monoclonal antibody trastuzumab (Herceptin). By labeling trastuzumab with a superparamagnetic iron-oxide nanoparticles (SPIO) a specific agent able to target cancer cells that overexpressed Her-2/neu could be designed though *in vivo* validation of the approach is still lacking (Smith, 2010; Artemov et al., 2003).

Tissue homeostasis is normally achieved by a tight regulation of proliferation, differentiation, and apoptosis. Apoptosis, or programmed cells death, is downregulated in cancer cells. A general therapeutic strategy may be therefore to induce apoptosis. Development of such treatments would benefit from imaging assays that specifically target molecular players involved in apoptotic signaling or cell surface marker that are specifically expressed on the surface of cells undergoing programmed cell death (Rudin, 2005b). For example, cells undergoing apoptosis redistribute aminophospholipids, primarily phosphatidylserine, to the outer layer of the cell membrane. Phagocytic cells, thus constituting a signal for cell removal, recognize exposed phosphatidylserines. Phosphatidylserine is recognized by peptidic molecules such as annexin-V and synaptogamin I. The latter has been labeled with SPIO nanoparticles and used *in vivo* as apoptosis-specific contrast agent. The nanoparticulate probe can leave the vascular bed in tumors since tumor vessels are immature and leaky, hence uptake is likely to be non-specific. Nevertheless it could be shown that the target specific probe was better retained in subcutaneously implanted tumors in mice while non-targeted SPIO nanoparticles were rapidly cleared from the tumor site (Zhao et al., 2001).

Molecular imaging can also be used as a complementary tool to monitor angiogenesis. In particular, it offers the possibility to differentiate angiogenic vessels from normal blood vessels by detecting differences in the expression of molecular markers (McDonald and Choyke, 2003). In the angiogenic cascade, different cell surface receptors, including the α_v_β_3_-integrin, are strongly expressed on activated endothelial cells. Mulder et al. (2005) have described the possibility to imaging angiogenesis using α_v_β_3_-specific bimodal lipidic nanoparticle both with MRI and fluorescence imaging.

The motivation for using MRI-based contrast agents, instead of other imaging modalities, is the possibility to combine together both the target-specific information with the high anatomical definition. Moreover, MRI is able to provide three-dimensional imaging which enables the possibility for an accurate quantification of the probe concentration, which otherwise is not be possible in the case of two-dimensional techniques as SPECT or optical imaging. The drawback of MRI approach is the low sensitivity, i.e., high local concentration of the reporter construct is required to induce detectable changes in the relaxation rates (Rudin, 2005b). In addition, MRI reporter molecules are in general bulky and may not easily reach the target site. However, for tumors this might be less an issue due to the leaky vasculature. Today, none of the MRI based target-specific probes has been approved for clinical use.

## MATHEMATICAL TOOLS FOR HANDLING MULTI-PARAMETRIC IMAGING DATA: A CLASSIFICATION PROBLEM

In each three-dimensional image dataset the object (tumor) is characterized by a set of voxels, with parameter values (features) that are characteristic for the respective measurement attributed to every voxel. Examples are values for the relaxation time, apparent water diffusion coefficient, or vascular permeability. Assuming that the dataset are properly coregistered all voxels *v*_x,y,z_ are characterized by a vector, whose elements are the parameter values *f*_i_ allocated to the various measurements, i.e.,

$$v_{x,y,z;t}=v_{x,y,z;t}\left(f_{1},f_{2},...,f_{N}\right).$$

The dimension of this data set is *D×T×N*, where *D* is the number of voxels, *T* the number of time points measured (*T* = 1 for static measurements) and *N* the number of features evaluated.

In mathematical terms these set of voxels (three-dimensional maps) form a dataset that contains all the information collected for the tumor. Although all the data are stored in a simple structure as a basic database, it is not easy to extract and quantify information from it. Usually, radiologists consider just few features and mentally divide the tumor in macro-regions, for which individual parameters are analyzed. Obviously this type of analysis discards many the majority of features contained in the dataset and the validity of conclusion critically depends on the experience of the reader. There is no way for human brain to systematically process all the available information voxel by voxel.

**FIGURE 8:**
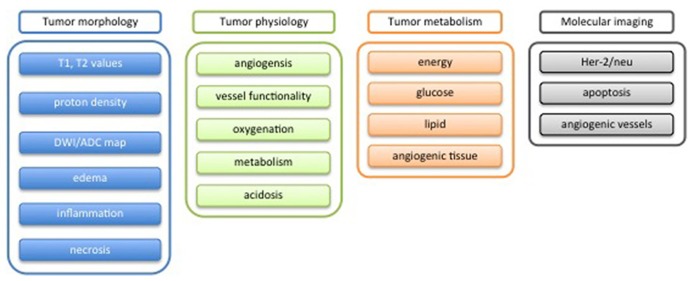
**Scheme for potential tumor phenotypic characterization by mean of MRI**.

The three-dimensional maps contain all measured information on the object reflecting both morphological aspects and physiological behavior. Information regarding the heterogeneity of the object is intrinsically contained. Taking into account this huge amount of information requires mathematical tools that allow a data reduction in a robust manner. One output of such tools is to classify each voxel of the tumor according to the measured features, and finally generate a map of the different tissues types present in the tumor. Several mathematical methods, which come from the field of information theory, have been developed for this purpose. A schematic workflow of the quantification process is shown in **Figure 8**.

### EXTRACTING OBJECT FEATURES FOR CLASSIFICATION

**FIGURE 9:**
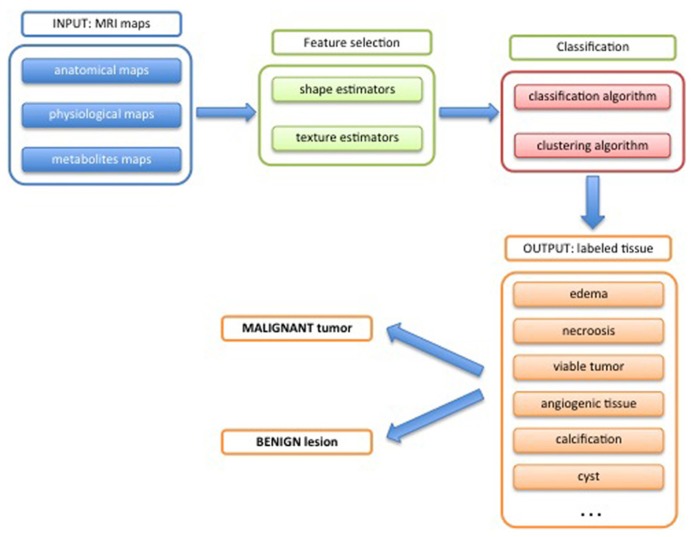
**Schematic workflow of the quantification process**.

As mentioned before, all the information are stored in a *dataset*, where the *features* are any kind of map (measured by MRI, **Figure 9**) and the *subject* are the individual voxels voxel of the three-dimensional matrix.

The first step of the classification process consists of the selection of useful features from the dataset. This process called *feature selection* aims at taking into account only features that contain significant and non-redundant information in order to minimize the confusion intrinsic noise of the data (Umbaugh, 2011). For this purpose different approaches, that describe the variability of the dataset, can be pursued. The traditional way, which comprises a set of techniques that perform a simultaneous statistical analysis of all features, is called *multivariate analysis*. Such techniques include multivariate analysis of variance (MANOVA), principal component analysis (PCA), factor analysis, multidimensional scaling, and correspondence analysis. All of them have as goal to determine a new set of synthetic variables that best represent the samples in a statistical interval.

Another approach consist of considering all the features and assign them a ranking score according to their discriminant power and accuracy, and then simply select the top ranked ones as final features used for the classification (Press et al., 2007; Zacharaki et al., 2009). These methods can be divided in three main categories: filter algorithm, wrapper, and embedded methods. For a comprehensive mathematical description of these methods the reader is referred to (Guyon and Elisseff, 2003).

Another important issue is the quantifications of the characteristics inherent in 3D feature maps. In other words, specific estimators that take into account the heterogeneity and the complexity of the object (tumor) are determined (Dominietto et al., 2012). Two types of estimators are commonly used: shape and texture estimators. The first group describes the geometry of the object (whole tumor or specific region), and extracts shape descriptors such as volume, surface area, compactness and signature (Rangayyan and Nguyen, 2007; Rangayyan et al., 2010). Texture estimators are related with the contents of the object and in particular to its texture by means of a set of estimators as fractal dimension (Lopes and Betrouni, 2009), lacunarity (Plotnick et al., 1996), Laws’ measures (Rangayyan, 2005), and Haralick’s measures (Haralick, 1979). Both shape and texture estimators also used in the geometrical segmentation of anatomical structure (Rangayyan, 2005).

### CLASSIFICATION

For classification two common techniques are currently used: pattern recognition and clustering technique (Umbaugh, 2010).

In general terms, for a given group of objects (i.e., different kinds of tissue or different type of tumors), pattern recognition algorithms aim at identifying the individual objects and assign them the correct label. In order to perform this operation, the algorithm has been “trained” previously with a dataset consisting of known objects (training dataset) by means of which it learns to recognize the objects from their features. This process is called supervised machine learning (Bishop, 2006).

The clustering approach is different as it does not require previous knowledge on the objects. Briefly, for analyzing multiparametric static data each voxel represent a subject in a *N*.*D*-dimensional space, where *N* is the number of features and *D* the number of voxels. Voxels that share similar properties will have similar features values and therefore will form a group (or cluster) of points in the *N*.*D* space. The objective of using the cluster algorithm is to identify the different groups of points (Theodoridis and Koutroumbas, 2006). The combination of features expressed by each group characterizes its morphological, physiological, metabolic, or molecular properties: it is therefore necessary, but not always straightforward, to translate the feature fingerprint into biomedically relevant information.

For both approaches, the most critical point is feature selection as subsequent tissue classification, e.g., differentiating tumor from healthy tissue or classifying subregions within a tumor, critically depends on the discriminative nature of the features.

Most of the studies to classify tumor tissues relate to brain tumors. Brain, in fact, offers many advantages related with the image acquisitions: easy positioning and fixation, absence of or minimal physiological movements, availability of several anatomical landmarks that renders co-registration rather straightforward in case of multi-modalities acquisitions. Different approaches have been described in the literature to classify and segment brain tumors using texture analysis (Qurat-Ul-Ain et al., 2010), neural networks (Arizmendi et al., 2012), linear discriminant analysis decision tree support vector machine (Zacharaki et al., 2009), and clustering (Jagadeesan and Sivanandam, 2013). Similar studies have been reported for breast tumor in order first to discriminate between malignant tumors and benign microcalcifications (Rangayyan and Nguyen, 2007; Mu et al., 2008), and second to classify tumor lesions (Zheng et al., 2007; Tang et al., 2009; Glasser et al., 2013).

## *IN VIVO* HISTOLOGY USING MRI/MULTIMODAL ANALYSIS: POTENTIAL AND ISSUES

Multiple features have to be evaluated in order to comprehensively characterize biological tissue. Histological analysis, the gold standard for such investigation, used morphological features as well as specific molecular markers to unambiguously identify a specific tissue type. Yet, histology is based on tissue specimen, which for diagnosis are typically obtained via biopsy. Standard biopsy involves focal sampling of only small portions of tissue, and hence carries the risk, that critical regions may be missed in particular when sampling highly heterogeneous tissue such as tumors. The possibility to acquire *in vivo* 3D multi-parametric information on tissues, in our context tumors, in a non-invasive manner might offer important benefits in management of cancer patients. Compared to biopsy, imaging (MRI) based tissue characterization allows analyzing the whole tumor yielding information over its entire volume thereby avoiding the problem of sampling errors. As the measurement is non-invasive, changes in tissue features can be monitored longitudinally, which is highly relevant for prognosis and for evaluating therapy response. The comprehensive nature of tissue analysis provided by imaging supports histological analysis by guiding biopsy sampling thereby minimizing the possibility of sampling errors.

An important advantage of the *in vivo* measurement is the possibility to study physiological processes, which evidently cannot be assessed *ex vivo*. Measurements of processes such as tumor angiogenesis, perfusion, metabolism, or oxygen consumption provide essential information for determining the stage of the tumor. Also it has been shown that such readouts may be early indicators of therapy response, proceeding morphological changes. Similar to morphological features, tumor physiological and metabolic parameters are highly heterogeneous, for example different tumor stages may coexist in the same proliferative mass in glioma patients (Zacharaki et al., 2009). Apart from spatial heterogeneity tumor physiology and metabolism also fluctuate over time (Bonadonna et al., 2007).

*In vivo* tissue characterization based on imaging has emerged as important tool for the detection and characterization of solid tumors including metastases (Mia, 2011). Today, MRI together with PET (Positron Emission Tomography), SPECT (Single Photon Emission Computer Tomography), CT (Computer Tomography), and US (Ultra Sound) provide a platform that provides multiparametric information characterizing tumor morphology, physiology, metabolism as well as cellular and molecular properties. These techniques are currently used in the clinic to gain as comprehensive information as possible before deciding the best treatment for the patient. Nevertheless, the evaluation of this huge amount of data is usually qualitative and relies on skills of the radiologist. A standardize quantitative evaluation, which gives robust and reproducible results is at the moment missing.

At present there is a huge diversity of imaging/MRI methods that are used in experimental animal studies that provide the multiparametric information required for using the classification tools. However, only a few are being used in the clinics, standard features derived from DCE, FLAIR, T1w, and T2w images and used as qualitative indicators of tumor stage (Young, 2007). More sophisticated techniques as DTI (Diffusion Tensor Imaging), MRS together with machine learning infrastructure, can provide complementary features that better characterize tumor physiology and micro-environment behavior, which would enhance the value of multiparametric analysis. It is important to introduce such method in a standardized manner into radiological practice.

Obviously MRI does not reach microscopic resolution; (Heyn et al., 2005; Martin, 2011), for *in vivo* experiments, the detection limit is in the range between 100 and 500 cells (Heyn et al., 2005; Muja and Bulte, 2009). This is relevant insofar, as final diagnosis is based on the cellular (type and shape) and molecular information (surface epitopes expressed by the cells) derived from histology. In order to reach this detail of information at the macroscopic level sampled by MRI, target specific contrast agents have to be used. We have seen, that such agents can be developed; yet there are substantial hurdles to overcome, before such agents will make it to the clinics. Scientific hurdles mainly relate to probe specificity and even more so probe delivery. MRI contrast agents are bulky and in general do not cross tissue barriers (membranes). Despite substantial efforts, this still constitute a major problem. The second hurdle relates to economics: development of such an agent is expensive. MRI probes are not administered in tracer amounts, which requires full safety and toxicology analysis. Multicenter clinical trials to demonstrate diagnostic relevance have to be carried. The complexity of developing MRI contrast agents to the market is reflected by the fact that only a very small number of generic agents is currently available for clinical use and it is unlikely that this is going to change in the near future. Hence, MRI methods to be used in clinical setting have to exploit endogenous contrast and rely on the contrast agents currently available. Nevertheless, together with spectroscopic readouts this already constitutes a fair basis for tissue characterization.

Multiparametric imaging based tumor characterization using morphological, physiological, metabolic – and eventually also cellular and molecular – features that can be monitored longitudinally in individual patients might open a way to personalization of the treatment. Today, for many tumor standard treatment protocols that are nevertheless tuned to the specific situation of each patient, are being pursued. This approach does not permit to exploit all possibilities offered today for tumor treatment. Highly specific drugs, new detailed reclassifications of tumor diseases, genetic characterization of several tumors as well as improvements in diagnostic technologies are dramatically changing the landscape of oncology toward patient-specific personalized treatments (Tursz et al., 2011). On the other hand, given the high genetic instability of tumors, it has been questioned whether such approaches are in fact viable (Gillies et al., 2012). Nevertheless, it is beyond doubt that the combined analysis of multi-parametric readouts will improve the diagnostic accuracy, which ultimately should translate into an improved management of cancer patients

## Conflict of Interest Statement

The authors declare that the research was conducted in the absence of any commercial or financial relationships that could be construed as a potential conflict of interest.
